# Supplementary material for: Causal Relationship between Aging and Anorexia Nervosa: A White-Matter-Microstructure-Mediated Mendelian Randomization Analysis
Source: Biomedicines. 2024 Aug 16;12(8):1874. doi: 10.3390/biomedicines12081874 (PMC11351342; doi:10.3390/biomedicines12081874)
Supplement: Supplementary file 1 [file biomedicines-12-01874-s001.zip › Supplementary Figure/Figure S9/Leaveoneout plot of MR analysis for ubm-b-1631 to AN.pdf]

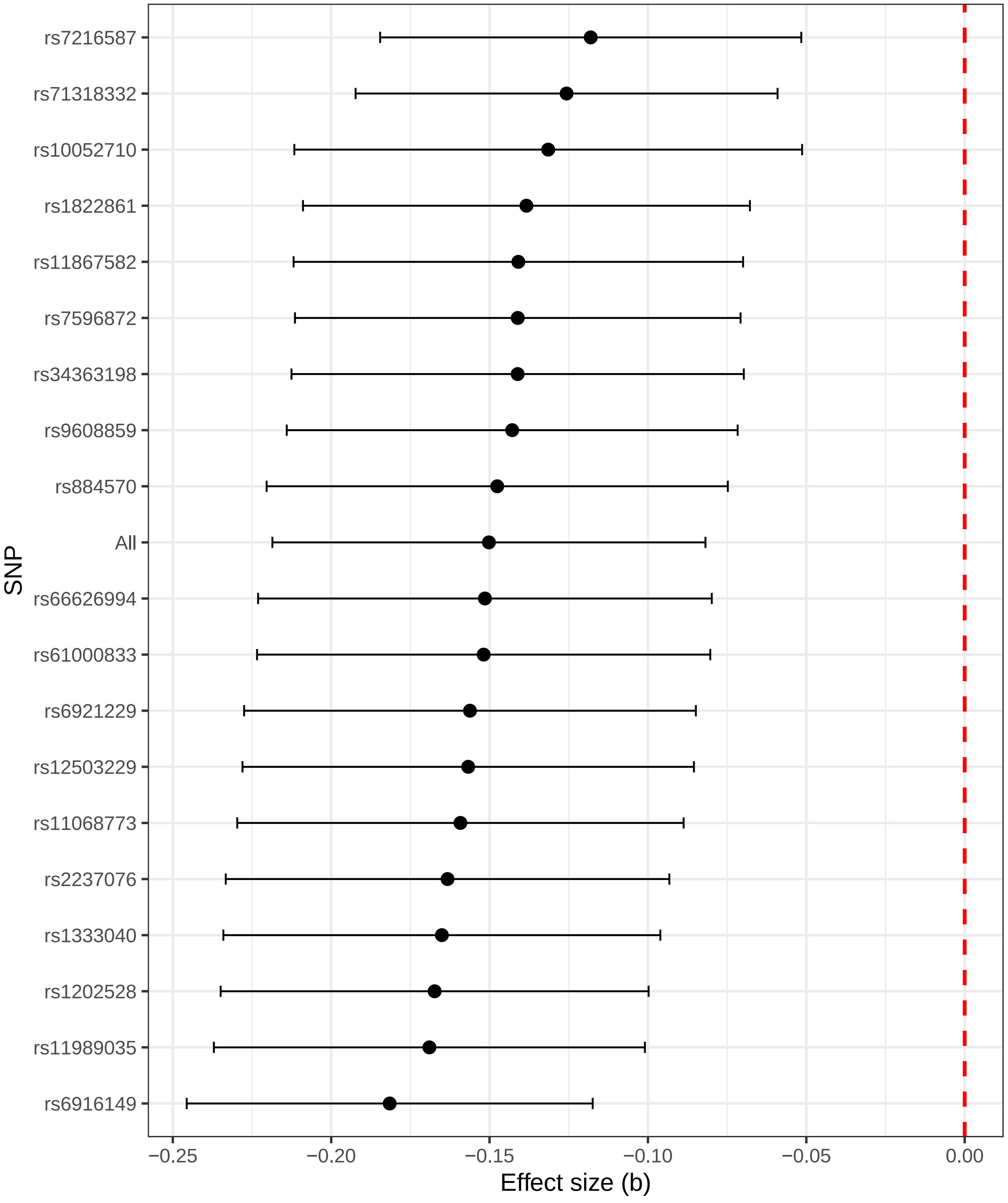

rs7216587
rs71318332
rs10052710
rs1822861
rs11867582
rs7596872
rs34363198
rs9608859
rs884570
All
rs66626994
rs61000833
rs6921229
rs12503229
rs11068773
rs2237076
rs1333040
rs1202528
rs11989035
rs6916149
SNP
−0.25
−0.20
−0.15
−0.10
−0.05
0.00
Effect size (b)
